# Toxic AGEs (TAGE) theory: a new concept for preventing the development of diseases related to lifestyle

**DOI:** 10.1186/s13098-020-00614-3

**Published:** 2020-11-30

**Authors:** Masayoshi Takeuchi

**Affiliations:** Department of Advanced Medicine, Medical Research Institute, Kanazawa Medical University, Uchinada-machi, Ishikawa, 920-0293 Japan

**Keywords:** Advanced glycation end-products (AGEs), Glyceraldehyde, Toxic AGEs (TAGE), Sucrose, High-fructose corn syrup (HFCS), Dietary AGEs, Lifestyle-related diseases (LSRD)

## Abstract

**Background:**

The habitual excessive intake of sugar (i.e., sucrose and high-fructose corn syrup), which has been implicated in the onset of diabetes mellitus, induces excessive production of glyceraldehyde, a metabolite produced during glucose and fructose metabolism, in hepatocytes, neuronal cells, and cardiomyocytes.

**Main text:**

Toxic advanced glycation end-products (toxic AGEs, TAGE) are formed from reactions between glyceraldehyde and intracellular proteins, and their accumulation contributes to various cellular disorders. TAGE leakage from cells affects the surrounding cells and increases serum TAGE levels, promoting the onset and/or development of lifestyle-related diseases (LSRD). Therefore, serum TAGE levels have potential as a novel biomarker for predicting the onset and/or progression of LSRD, and minimizing the effects of TAGE might help to prevent the onset and/or progression of LSRD. Serum TAGE levels are closely related to LSRD associated with the excessive ingestion of sugar and/or dietary AGEs.

**Conclusions:**

The TAGE theory is also expected to open new perspectives for research into numerous other diseases.

## Background

Research on protein glycation (the Maillard reaction) began with the discovery of melanoidin by Maillard in 1912 [1]. The Maillard reaction was initially regarded as a browning reaction in the food chemistry field, and its effects on the taste of food have been studied. In 1968, hemoglobin A1c (HbA1c), an early glycation product, was first detected in the human body [2], while advanced glycation end-products (AGEs) were discovered by Cerami et al. in the 1980s [3]. The receptor for AGEs (RAGE) was cloned by Neeper and Schmidt et al. in 1992 [4], and RAGE transgenic mice were produced by Yamamoto et al. in 2001 [5]. AGE molecules, such as Nε-(carboxymethyl)lysine (CML) [6], pyrraline [7], and pentosidine [8], were subsequently identified, and the majority of anti-AGE antibodies used in 1996 were shown to recognize the CML structure [9]. Therefore, the concept that CML is the main structure of AGEs has spread worldwide.

However, research into various anti-AGE antibodies by our group has demonstrated that AGE structures other than CML are more closely associated with clinical parameters [10]. We reported the concept of non-CML AGEs in 1999 [10], subsequently identified a non-CML AGE that exhibited strong cytotoxicity [11, 12], and proposed the hypothesis that “toxic AGEs (TAGE)” contribute to lifestyle-related diseases (LSRD) in 2004 [13].

The significance of serum TAGE levels as a new biomarker that could aid the early diagnosis and prevention of LSRD and evaluations of treatment efficacy are described herein. Furthermore, whether limiting sugar and/or dietary AGE intake decreases the production or accumulation of TAGE, and hence, prevents the onset and/or progression of LSRD, is discussed.

## AGE generation in the human body

The balanced intake of carbohydrates, proteins, lipids, vitamins, and minerals is necessary to maintain health. Glucose, a carbohydrate, cannot function properly unless it is present in the blood at appropriate levels, and protein glycation occurs continuously in the human body. HbA1c, which is used in diagnostic tests for diabetes mellitus (DM), is one of the early glycation products generated by the reaction of glucose with hemoglobin in erythrocytes [2]. AGEs are generated under hyperglycemic conditions [14–17]. We reported that α-hydroxyaldehydes (glyceraldehyde [GA] and glycolaldehyde), dicarbonyl compounds (glyoxal [GO], methylglyoxal [MGO] and 3-deoxyglucosone [3-DG]), and also fructose contribute to protein glycation [10, 11, 18, 19].

**Fig. 1:**
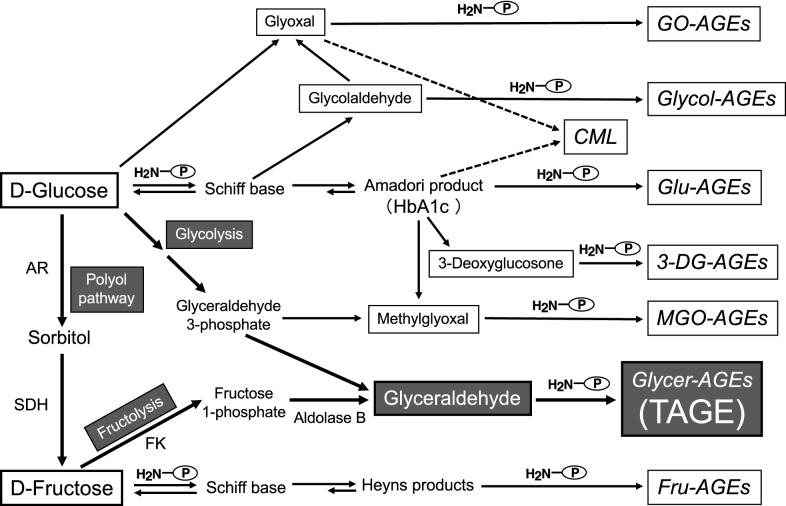
Alternative in vivo AGE generation routes. Reducing sugars, such as glucose, fructose, and glyceraldehyde, react non-enzymatically with the amino groups of proteins to form reversible Schiff bases and Amadori products/Heyns products. These early glycation products undergo further complex reactions, such as rearrangement, dehydration, and condensation, to become irreversibly cross-linked, heterogeneous fluorescent derivatives, termed AGEs. HbA1c: hemoglobin A1c; CML: Nε-(carboxymethyl)lysine; GO-AGEs: glyoxal-derived AGEs; Glycol-AGEs: glycolaldehyde-derived AGEs; Glu-AGEs: glucose-derived AGEs; 3-DG-AGEs: 3-deoxyglucosone-derived AGEs; MGO-AGEs: methylglyoxal-derived AGEs; Glycer-AGEs: glyceraldehyde-derived AGEs; TAGE: toxic AGEs; Fru-AGEs: fructose-derived AGEs; AR: aldose reductase; SDH: sorbitol dehydrogenase; FK: fructokinase; P-NH_2_: free amino residue of a protein

Seven immunochemically distinct classes of AGEs (glucose-derived AGEs [Glu-AGEs], fructose-derived AGEs [Fru-AGEs], glycolaldehyde-derived AGEs, GA-derived AGEs [Glycer-AGEs], MGO-derived AGEs, GO-derived AGEs, and 3-DG-derived AGEs) have been detected in sera from hemodialysis patients with diabetic nephropathy (DN-HD) [10, 11, 18, 19]. Thus, Maillard reaction, sugar autoxidation, and sugar metabolic pathways (glycolysis/the polyol pathway/fructolysis) have been proposed to be involved in AGE formation in vivo (Fig. 1). Glycer-AGEs formed from GA, a trisaccharide (triose sugar) intermediate of fructose and glucose metabolism, exhibit strong cytotoxicity [12]; therefore, we proposed the novel concept of TAGE [13]. TAGE are generated from digested starch, the main component of rice, bread, and noodles, as well as metabolites of the sugar (sucrose and high-fructose corn syrup, HFCS) added to beverages and processed foods, and fluctuations in TAGE levels are closely related to dietary habits in humans (Fig. 1).

## Concepts of non-toxic AGEs and TAGE

**Fig. 2:**
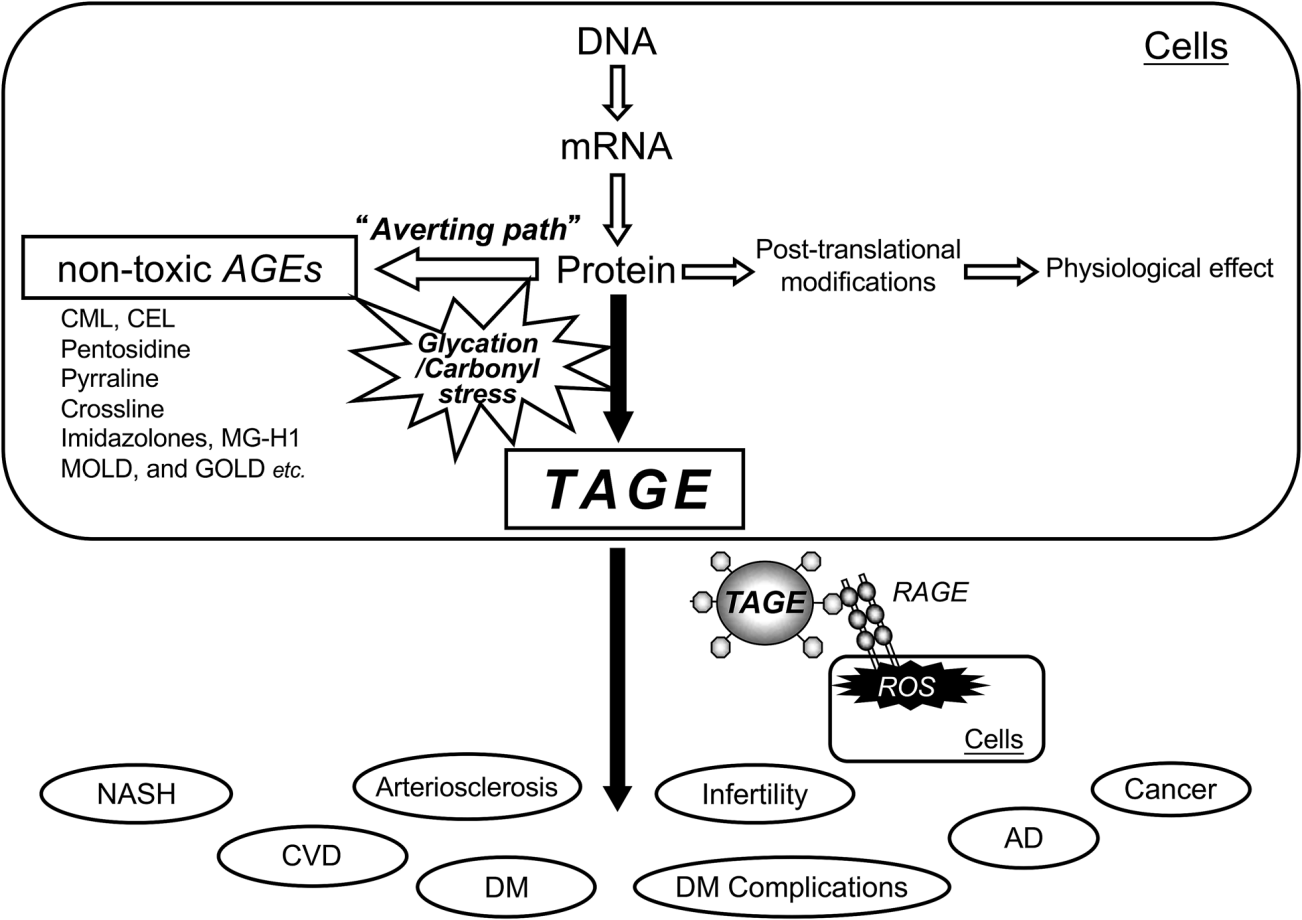
The TAGE theory of LSRD. Among the various types of AGE structures generated in vivo, TAGE, but not non-toxic AGEs, such as CML, CEL, pentosidine, and pyrraline, appear to play an important role in the pathophysiological processes associated with LSRD. We postulate that non-toxic AGE structures may be physiologically relevant for preventing the potentially damaging consequences of the advanced glycation process. CEL: Nε-(carboxyethyl)lysine; MG-H1: methylglyoxal-hydroimidazolone; MOLD: methylglyoxyl-derived lysine dimer; GOLD: glyoxyl-derived lysine dimer

It remains unclear why many types of AGEs are generated in the human body. Based on our research, in vivo AGE-generating reactions are physiologically significant because they induce post-translational protein modifications. Proteins, which are translated according to the genetic information in DNA, are responsible for various physiological processes involving post-translational modification reactions. However, in the presence of “glycation/carbonyl stress” and increased production of aldehyde/carbonyl compounds in vivo intracellular proteins can non-enzymatically react with such compounds to produce various AGEs. The generation of non-toxic AGEs, such as CML, pentosidine, and pyrraline, which do not exert direct cytotoxic effects, is a biological defense mechanism, in which proteins actively trap aldehyde/carbonyl compounds with high chemical reactivity to detoxify them. Ahmed et al. [20], who first identified CML, reported that CML is generated through an averting path. Similarly, the process that is responsible for the generation of non-toxic AGEs might be involved in the detoxification of most end-products of glycation/carbonyl stress in the human body. However, TAGE have been implicated in the pathogenesis of DM and associated vascular complications, as they bind to RAGE [21–23]. Interactions between extracellular TAGE and RAGE induce reactive oxygen species (ROS) generation in numerous types of cells [23]. TAGE have also recently been implicated in cardiovascular disease (CVD), non-alcoholic fatty liver disease (NAFLD)/non-alcoholic steatohepatitis (NASH), arteriosclerosis, infertility, Alzheimer’s disease (AD), and cancer [21–31] (Fig. 2).

More recently, we demonstrated that intracellular TAGE formation and accumulation induced not only neuronal cell damage [32], but also hepatocellular damage [33–35] and pancreatic ductal epithelial cell damage [36], cardiomyocyte pulsation arrest and cell death [37], and myoblast cell death [38]. Therefore, TAGE are accumulate in cells, cause cell damage, and leak extracellularly into the blood, thereby increasing TAGE levels in circulating fluids.

## Cytotoxicity of TAGE in the liver

According to the clinical practice guidelines for NAFLD/NASH, overeating and a lack of exercise cause non-alcoholic fatty liver (NAFL) and insulin resistance (IR), and various factors then contribute to the progression of NASH, resulting in fibrosis and potentially cirrhosis, liver failure, and hepatocellular carcinoma (HCC) [39, 40]. Our group focused on the TAGE-RAGE axis. We reported that the addition of TAGE to hepatocytes increased IR and C-reactive protein (an inflammatory marker) levels [41, 42], while the expression levels of fibrotic markers, such as collagen type Iα2, transforming growth factor-β1 (TGF-β1) and monocyte chemoattractant protein-1 (MCP-1), were upregulated in hepatic stellate cells (HSC) [43]. We recently revealed that hepatocytes underwent necrosis and TAGE formation induced by caspase 3, which is involved in cell death [31, 34]. As a result, TAGE leaked out of the cells, affecting the surrounding hepatocytes and HSC through RAGE.

Thus, we examined the potential of TAGE leakage from damaged hepatocytes into the blood as a diagnostic marker. Serum TAGE levels were determined using a competitive enzyme-linked immunosorbent assay (ELISA) method, involving an anti-TAGE-specific antibody developed by our group [11, 44, 45]. The epitope recognized by the anti-TAGE antibody is different from the previously reported GA-derived AGE structures; i.e., 3-hydroxy-5-hydroxymethyl-pyridinium compound (GLAP) [46] and triosidine [47]. We found that there were differences between the anti-TAGE antibody and the antibodies against well-defined AGEs as well as the antibodies against AGEs formed from reducing sugar/carbonyl molecules with unknown structures [International patent application for anti-TAGE antibody: PCT/JP2019/34,195].

## Serum TAGE levels in NAFLD/NASH

### NASH

NAFLD ranges from NAFL to NASH and is one of the most common causes of hepatic disease throughout the world. We assessed the serum AGE levels (TAGE, CML, and Glu-AGEs) in 66 patients that had been diagnosed with histologically verified NASH without any evidence of liver cirrhosis, 10 patients with NAFL, and 30 healthy controls [48]. The NASH patients (9.8 ± 3.7 U/mL) exhibited greater TAGE accumulation in both tissue and serum than the NAFL patients (7.2 ± 2.3 U/mL) and healthy controls (7.0 ± 2.4 U/mL). There was a positive correlation between serum TAGE levels and the homeostatic model assessment of IR (HOMA-IR), and there was an inverse association between serum TAGE levels and adiponectin levels. TAGE were also detected in the hepatocytes of NASH patients, but negligible TAGE levels were seen in NAFL patients, and no significant differences in CML or Glu-AGE levels were observed between these groups [48].

We demonstrated that hydroxymethyl-glutaryl-CoA reductase inhibitor atorvastatin reduced the serum TAGE levels in 43 biopsy-proven NASH patients with dyslipidemia [49]. Following treatment with atorvastatin for 6 months (at 10 mg/day), significant reductions in liver alanine aminotransferase and γ-glutamyl transpeptidase (γ-GTP) activity were seen in all patients. In addition, the patients’ plasma adiponectin levels were increased while their plasma tumor necrosis factor-α levels were decreased after the treatment. The patients’ serum TAGE levels decreased significantly during the treatment (before vs. after: 10.4 ± 3.8 vs. 5.9 ± 3.3 U/mL, respectively).

Conversely, the generation and accumulation of TAGE were detected in hepatocytes cultured under high-fructose conditions [50] and in the livers of rats reared on a high fructose and high fat diet [51] or with 10%-HFCS water [52]. Therefore, the habitual excessive intake of fructose (i.e., HFCS/sucrose/100% juices) was shown to support the formation/accumulation of TAGE in the human body.

### Non-B or non-C (NBNC) HCC and rectal cancer

We previously showed that NBNC-HCC patients had significantly higher serum TAGE levels (18.2 ± 5.4 U/mL) than NASH patients without HCC (10.4 ± 3.4 U/mL) and control subjects (7.0 ± 2.4 U/mL) [53]. Multiple regression analysis demonstrated that age and γ-GTP and high-density lipoprotein cholesterol (inversely) levels were significantly and independently associated with TAGE levels.

The European Prospective Investigation into Cancer and Nutrition (EPIC), which was mainly conducted in Europe and the United States, revealed that the high serum TAGE group (median value: 10.3 ± 1.7 U/mL) was at higher risk of rectal cancer after 4 years (odds ratio (OR): 1.90; 95% confidence interval [CI] 1.14–3.19). Additionally, the risk of rectal cancer was even higher (OR: 2.70; 95%CI 1.29–5.62) in humans with a drinking habit (median total alcohol intake of controls: males: 18.1 g/day; females: 5.7 g/day) [54].

## Sucrose/HFCS and LSRD

Previous studies have shown that the long-term consumption of high quantities of sugar also contributes to the onset and/or development of NASH, CVD, and AD; however, the responsible molecular mechanisms are yet to be discovered [55–60]. We demonstrated a strong correlation between TAGE levels and LSRD, and the habitual intake of high quantities of sugar-sweetened beverages (SSB) elevated hepatic GA levels. Increasing epidemiological and mechanistic evidence indicates that the effects of consuming high levels of sugar on human health cannot be explained by a simple increase in calories [61]. Sugar is known to be involved in the pathogenesis of metabolic syndrome-related diseases [62, 63], such as NAFLD/NASH, DM, and CVD, as well as the aging process, which is accelerated by glycation-related protein damage [14–17].

### Sugar contents of commercial beverages

The American Heart Association recommended that most Americans should reduce their added sugar intake to ≤ 100 kcal-150 kcal/day (females: 25 g/day; males: 37.5 g/day) [64]. However, these values are greatly exceeded in most regions, and thus, since sugar has the same harmful effects as alcohol, sugar intake needs to be regulated [65]. The 2015 World Health Organization (WHO) guidelines recommended a reduction in the daily intake of added sugar to < 5% of total energy intake by adults and children (i.e., 25 g sugar for a 2000 kcal/day diet) to obtain additional health benefits [66]. We examined the sugar contents of beverages and found that approximately 40% of the 885 types of commercially available beverages in Japan contained > 25 g sugar [67].

### Restricting SSB consumption

A 500-ml bottle of a carbonated beverage, such as Coke, Sprite, or Fanta, contains 50–60 g of added sugar, which exceeds the recommended daily amount of added sugar. Therefore, the WHO recommended the taxing of SSB in October 2016 [68].

We showed that in Goto-Kakizaki rats, a rodent DM model, serum TAGE levels were significantly decreased after 6 weeks’ treatment with nateglinide (a rapid-acting insulin secretagogue; 50 mg/kg, twice daily just before each meal) [69]. We also found that the serum TAGE levels, but not the HbA1c or Glu-AGE levels, of DM patients were significantly decreased after 12 weeks’ treatment with acarbose (an α-glucosidase inhibitor, 150 mg/day) [70]. These findings indicated that the habitual excessive intake of carbohydrates promotes the generation/accumulation of TAGE in the human body.

## Dietary AGEs and LSRD

Two major types of AGEs, endogenous and exogenous AGEs, have been identified in humans [71, 72]. We observed elevated expression of RAGE and vascular endothelial growth factor (VEGF) in liver and accelerated TAGE formation and accumulation in normal rats that were given AGE-rich beverages in their diet [73].

### AGE contents of beverages and processed foods

We assessed the concentrations of various AGEs in 885 types of beverages and 767 types of processed food that are frequently consumed in Japan. The levels of four AGEs (CML, Glu-AGEs, Fru-AGEs, and TAGE), which have been detected in the serum samples of non-DM and DM subjects, were determined employing competitive ELISA involving specific immunopurified antibodies [10, 11, 18, 19]. The assays showed that Glu-AGEs and Fru-AGEs, but not CML or TAGE, were present in considerable concentrations in the beverages and processed foods. Glu-AGEs, Fru-AGEs, CML, and TAGE were detected at levels of ≥ 85%, 2–12%, < 3%, and trace amounts, respectively, in the beverages and at levels of ≥ 82%, 5–15%, < 3%, and trace amounts, respectively, in the processed foods [74].

### Restricting AGE consumption

In humans, roughly 10% of dietary AGEs (measured as the levels of CML in serum and urine after the ingestion of an AGE-containing meal) are taken into the body; one-third is eliminated by urinary excretion within 48 h of intake, and two-thirds are retained within the body [75]. We found that the serum Glu-AGE levels of healthy subjects and DN-HD ranged from 10 to 20 and from 30 to 50 U/mL, respectively. Three months’ treatment with the oral charcoal-based drug Kremezin (6 g/day) significantly decreased the serum levels of Glu-AGEs (44.1 ± 10.8 vs. 27.6 ± 6.0 U/mL) and TAGE (13.2 ± 4.4 vs. 6.2 ± 0.9 U/mL) in non-DM patients with chronic renal failure (CRF), whereas these levels remained unchanged in age- and renal function-matched CRF patients who did not receive Kremezin [76]. In addition, the Kremezin-treated patients had significantly lower serum mRNA levels of RAGE and MCP-1 and exhibited significantly lower vascular cell adhesion molecule-1 (VCAM-1) expression on their endothelial cells (EC) [76].

Therefore, restricting the intake of SSB/processed food-derived sugars and dietary AGEs represents a novel strategy for suppressing the generation and accumulation of TAGE and preventing LSRD.

### Intracellular TAGE generation and accumulation

Using a neuronal culture system, we confirmed that TAGE are strongly neurotoxic [12, 77]. In addition, while an anti-TAGE antibody suppressed the neurotoxic effects of serum AGEs from DN-HD, no such effects were seen with antibodies against other AGEs or CML [12, 77]. In AD brains, TAGE were mostly found in the cytosol of neuronal cells in the hippocampus and parahippocampal gyrus, but TAGE were not present in senile plaques or astrocytes [78]. We also showed that intracellular TAGE production reduced amyloid β levels and increased total tau and p-tauT181 levels in the culture media and the intracellular levels of AD biomarkers (i.e., total tau, p-tauT181, VEGF, and TGF-β) in human neuroblastoma SH-SY5Y cells [32].

We revealed that intracellular TAGE generation/accumulation damages neurons, hepatocytes, pancreatic ductal epithelial cells, cardiomyocytes, and myoblast cells [32–38]. The TAGE precursor GA is generated in these cells, particularly hepatocytes, via three pathways [79]: (i) Glucose is metabolized glycolytically to GA-3-phosphate, before being non-enzymatically dephosphorylated and degraded to GA; (ii) fructose is metabolized to GA via a pathway involving fructokinase and aldolase B (fructolysis); and (iii) in hyperglycemic conditions glucose is metabolized to fructose via the polyol pathway, which regulates aldose reductase and sorbitol dehydrogenase, and the resultant fructose is metabolized to GA via fructolysis.

The liver plays a major role in carbohydrate homeostasis, controlling glucose levels by synthesizing and degrading glycogen and making glucose via gluconeogenesis [80]. The liver is also generally assumed to be the primary site of dietary fructose metabolism, and fructose promotes hepatic lipid accumulation via GA. Western foods that are rich in solid fats, fatty meals, full-fat dairy products, and highly processed foods tend to be the richest dietary sources of AGEs [81]. The thermal treatment of foods might increase their digestibility, nutritional value, and shelf-life. On the other hand, the AGE-modification of essential amino acids, particularly that of lysine, might reduce the nutritional value of proteins since cross-linked proteins are less digestible [82].

**Fig. 3:**
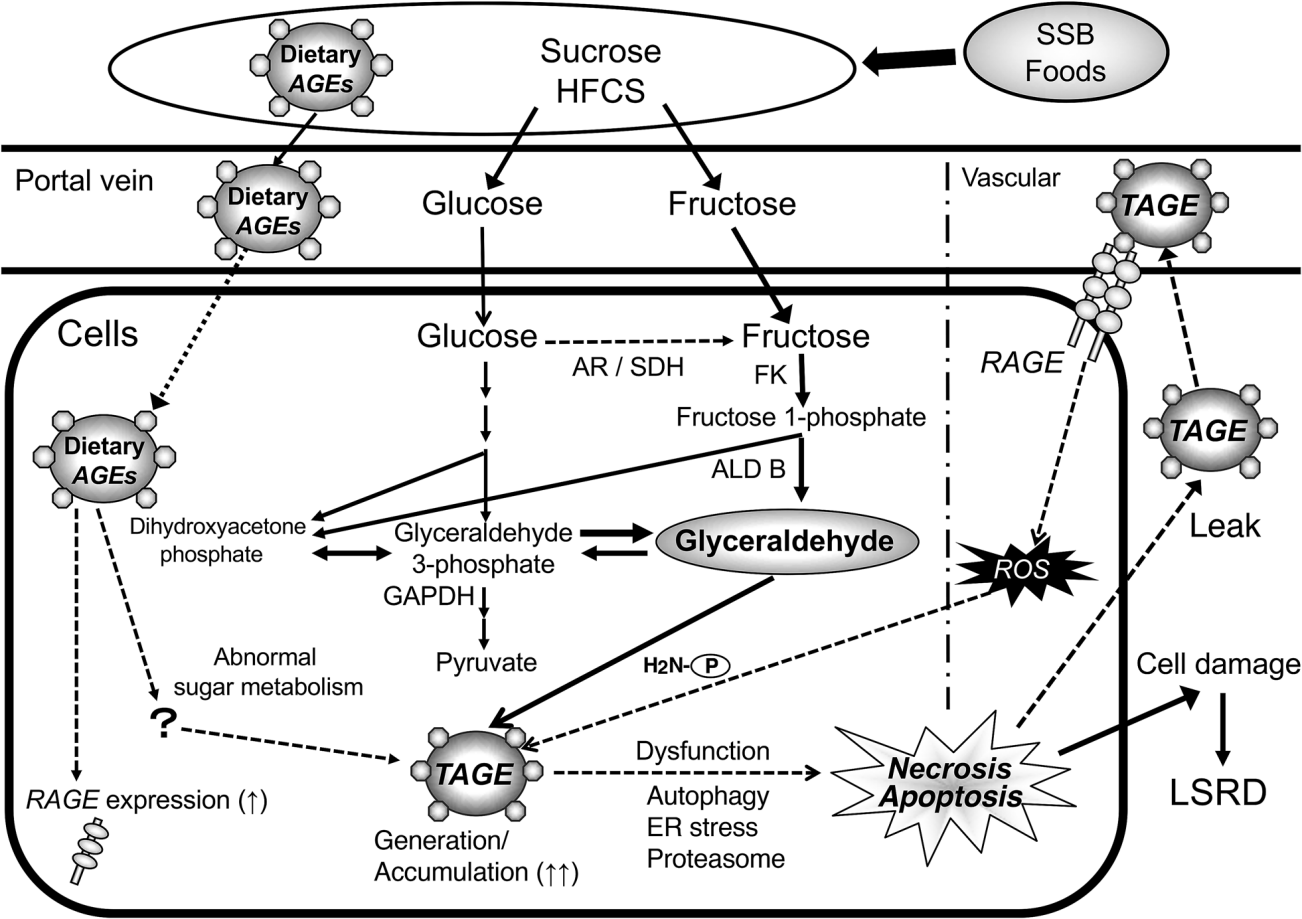
In vivo TAGE generation routes. The chronic intake of excessive amounts of SSB/processed foods increases the cellular levels of the sugar metabolite GA, which induces TAGE generation from intracellular proteins. Consequently, TAGE accumulate in cells, causing cell damage, and leak into the blood, increasing circulating TAGE levels. The TAGE-RAGE axis produces ROS, which appear to upregulate RAGE expression and TAGE generation. AR: aldose reductase; SDH: sorbitol dehydrogenase; FK: fructokinase; ALD B: aldolase B; GAPDH: glyceraldehyde-3-phosphate dehydrogenase; ER: endoplasmic reticulum; LSRD: lifestyle-related diseases; P-NH_2_: free amino residue of a protein

The habitual intake of large amounts of sugar and dietary AGEs [67, 74], which is characteristic of the modern diet, disturbs the metabolic system in hepatocytes; induces excessive GA production, which leads to TAGE being generated from intracellular proteins; and upregulates RAGE expression. Therefore, the accumulation of TAGE in cells leads to cell damage, which can allow TAGE to escape into the blood, thereby increasing circulating TAGE levels. Interactions between extracellular TAGE and RAGE alter intracellular signaling, gene expression, and the release of pro-inflammatory molecules and also induce ROS production in several cell types [23, 29], any of which could lead to the pathological alterations seen in LSRD (Fig. 3).

TAGE levels reflect the effects of not only blood glucose but also fructose and dietary AGEs, which are not reflected by blood glucose levels. Therefore, suppressing the effects of TAGE represents a novel strategy for preventing LSRD.

## Serum TAGE levels and LSRD

Serum TAGE levels could be useful as a new biomarker for diagnosing LSRD early or evaluating the effectiveness of measures to prevent/treat the onset and/or development of LSRD, regardless of the presence/absence of DM.

### Infertility

AGEs accumulate in DM patients and play an important role in the pathogenesis of the condition. Polycystic ovary syndrome, which is similar to DM, and aging are common causes of infertility. The associations between the serum TAGE concentration and the number of collected oocytes or rates of pregnancy were studied in people suffering from infertility. Both of these factors decrease with age, and even younger women with elevated serum TAGE levels (> 7.24 U/mL) experience reduced ongoing pregnancy rates [44]. Moreover, in women who are treated with assisted reproductive technologies (ART), correlations between serum TAGE levels and follicle development, fertilization, embryo development, and pregnancy were observed in poor responders, which suggested that TAGE accumulation is a valuable marker of fertility status that is not dependent on age or day-3 follicle-stimulating hormone levels [44].

In non-pregnant poor responders who were given sitagliptin, a dipeptidyl peptidase-4 (DPP-4) inhibitor, and underwent ART, ovarian dysfunction was ameliorated, and ongoing pregnancy rates increased significantly, in those patients whose serum TAGE levels were decreased by the sitagliptin. Pregnancy rates, both ongoing and clinical, were considerably higher in the sitagliptin-treated patients (20% and 14%, respectively) than in the controls (2.3% and 0%, respectively) [unpublished data]. Therefore, TAGE levels might be a valuable indicator that could aid the early diagnosis of ovarian dysfunction. In addition, reducing TAGE accumulation might be a novel therapeutic approach against poor ovarian responses.

### CVD

Endothelial progenitor cells (EPC) contribute to the maintenance of endothelial structure and function, and thus, promote vascular repair and angiogenesis. Even among healthy subjects with normal blood test values, reductions in the number and activity of vascular EPC were observed in a group with high serum TAGE levels. High serum TAGE levels (> 9.20 ± 1.85 U/mL) were found to be independently associated with a reduced number and decreased migratory activity of circulating EPC in otherwise healthy volunteers (34.6 ± 6.9 years old, 40 males and 8 females) [83], suggesting that TAGE impair EC repair. In healthy volunteers (53.7 ± 7.2 years old, 15 males and 15 females) that were administered collagen tripeptide (2.4 g/day for 6 months), which inhibits TAGE formation, the cardiac-ankle vascular index, an index of blood vessel stiffness, decreased with the serum TAGE level (9.95 ± 2.96 vs. 9.25 ± 2.82 U/mL) [84]. Thus, there is a possibility that serum TAGE levels are useful as a novel biomarker not only of vascular damage, but also for predicting future cardiovascular events. Further longitudinal studies are needed to clarify whether reducing the TAGE burden using AGE inhibitors and/or restricting the consumption of sugars/dietary AGEs could protect against CVD in non-DM/DM patients.

We also demonstrated that: (i) serum TAGE levels, but not those of HbA1c or CML, were independently related to vascular inflammation, as demonstrated by [^18^F] fluorodeoxyglucose-positron emission tomography, in outpatients [45]; (ii) among pre-DM patients, circulating TAGE levels were significantly higher in the high mean amplitude of glycemic excursions (MAGE) group than in the low MAGE group [85]; and (iii) an association between elevated baseline TAGE levels and plaque progression in the assessment of pitavastatin and atorvastatin was found in an acute coronary syndrome trial (The JAPAN-ACS Sub-study) [86].

### Non-DM/DM

DM patients were reported to have higher serum TAGE levels than healthy controls [87, 88]. We also demonstrated that serum TAGE levels, but not those of HbA1c, CML, or Glu-AGEs, were related to thrombogenic marker [89, 90], low-density lipoprotein cholesterol [91], serum pigment epithelium-derived factor [92], and DPP-4 [93] levels in the general population. Furthermore, we found that circulating TAGE levels were independently correlated with the HOMA-IR index in control subjects without DM [94]. In addition, serum TAGE levels were found to be (i) associated with visceral and subcutaneous adipose tissue inflammation and reduced adiponectin levels in outpatients [48, 95]; (ii) elevated in chronic kidney disease (CKD) and DM and correlated with the levels of inflammatory biomarkers, such as MCP-1 [88], the soluble form of VCAM-1 [96], and asymmetric dimethylarginine [97, 98]; and (iii) correlated with the soluble RAGE level, which might reflect RAGE expression in tissues, in non-DM/DM subjects [87, 88, 99–102], suggesting that the serum TAGE level is a useful marker of TAGE-RAGE axis activation.

We observed that the circulating TAGE levels in DM patients significantly decreased (11.9 ± 3.0 vs. 8.2 ± 0.8 U/mL) after 12 weeks’ acarbose treatment [70]. We also reported that a DPP-4 inhibitor [103], sulfonyl urea [104], and insulin [105] significantly reduced serum TAGE levels, and these reductions were found to be related to decreased levels of biomarkers of organ damage in DM and CKD patients. Moreover, we demonstrated that atorvastatin reduced the serum TAGE levels of DM/non-DM CKD/acute myocardial infarction patients [106–108].

Serum TAGE levels might be useful for identifying high-risk patients and provide valuable information for making treatment-related decisions.

## Conclusions and perspectives

**Fig. 4:**
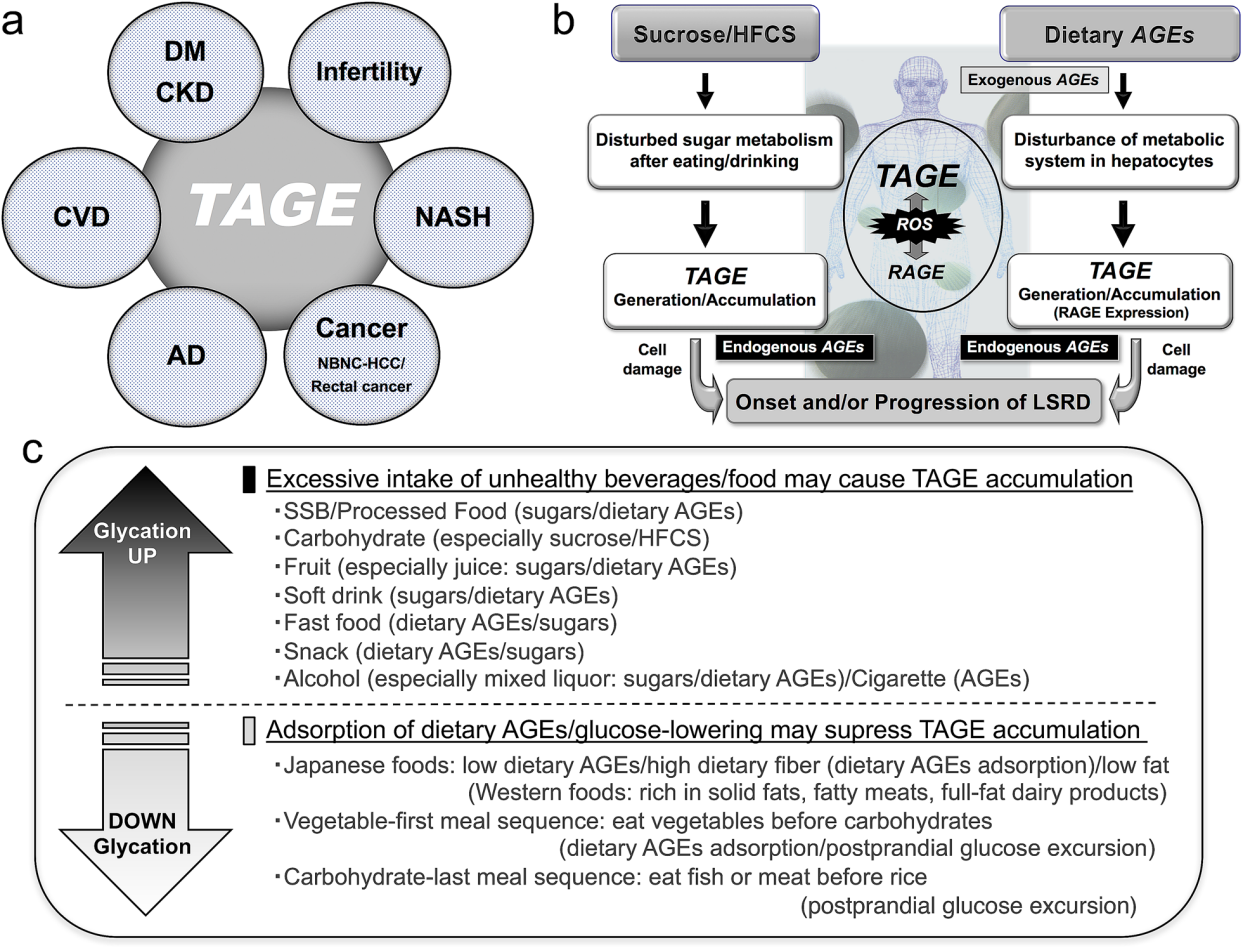
Conclusions/perspectives. **a** TAGE are a novel target for preventing the onset and/or progression of LSRD. **b** The onset and progression of LSRD are associated with habitual excessive intake of sugars/dietary AGEs. **c** Suppressing the generation/accumulation of TAGE may prevent the onset and/or progression of LSRD. The following strategies are recommended: (i) avoid habitual excessive intake of unhealthy beverages and/or food; (ii) increase the habitual intake of Japanese foods that suppress the generation/accumulation of TAGE; and (iii) adopt a vegetables-first and/or carbohydrates-last eating order to reduce rapid increases in blood sugar levels. The maintenance of dietary habits, as described above, represents a novel strategy for achieving a healthy and long life by suppressing the generation/accumulation of TAGE in the body, which contributes to the prevention of LSRD. AD: Alzheimer’s diseases; CKD: chronic kidney disease; CVD: cardiovascular disease; DM: diabetes mellitus; HFCS: high-fructose corn syrup; LSRD: lifestyle-related diseases; NASH: nonalcoholic steatohepatitis; RAGE: receptor for AGEs; ROS: reactive oxygen species; SSB: sugar-sweetened beverages; TAGE: toxic AGEs

The production and accumulation of TAGE in the human body, which are promoted by modern dietary habits, have important health implications. Even in healthy subjects with normal blood test values, high serum TAGE levels predict the onset and/or development of various LSRD. Therefore, the serum TAGE level might be a useful biomarker for aiding the prevention/early diagnosis of LSRD or evaluating the efficacy of treatments for LSRD (Fig. 4a, b).

Collectively, these findings indicate the importance of suppressing the formation and accumulation of TAGE in the human body by reducing the habitual excessive intake of sugar/dietary AGEs, as a novel way of preventing LSRD (Fig. 4c, upper). Western foods that are rich in solid fats, fatty meats, full-fat dairy products, and highly processed foods (mainly grilled, fried, deep fat fried, and roasted dishes) tend to be the richest dietary sources of AGEs [81]. On the other hand, the Japanese diet includes many low-fat foods, such as rice, seaweed, mushrooms, soy-based foods; i.e., tofu, and vegetables (especially root vegetables that are rich in insoluble/indigestible dietary fiber), and meals with low dietary AGE levels (mainly boiled and steamed dishes) [74].

The accumulation of TAGE in the kidneys might contribute to the progressive alteration of the renal architecture and the loss of renal function, such as mesangial cell and podocyte damage, in patients and rodents via various mechanisms [23]. Regularly consuming Japanese foods with low AGE levels and large amounts of insoluble dietary fiber, which absorbs and removes dietary AGEs, suppresses TAGE accumulation. The protective renal effects of Kremezin are considered to be due to its TAGE-lowering effects, which are based on the inhibition of dietary AGE absorption [76]. Therefore, the inhibition of sugar digestion and/or dietary AGE absorption by insoluble dietary fiber is a potentially useful novel strategy for preventing LSRD, such as CVD, DM, and CKD.

A vegetables-first [109, 110] and carbohydrates-last meal sequence [111, 112], followed by physical activity to promote the utilization of glucose have been recommended to prevent postprandial hyperglycemia. The consumption of vegetable or meat/fish dishes before carbohydrate dishes has also been shown to markedly improve postprandial glucose excursion in individuals with type-2 DM and healthy volunteers [109–112] (Fig. 4c, lower).

In addition to maintaining healthy dietary habits, suppressing the production and accumulation of TAGE in the body will help to prevent LSRD. The TAGE theory is also expected to open new perspectives for research into numerous other diseases.

## Data Availability

Not applicable.

## Abbreviations

95%CI: 95% confidence interval
AD: Alzheimer’s disease
AGEs: Advanced glycation end-products
ART: Assisted reproductive technology
CKD: Chronic kidney disease
CML: Nε-(Carboxymethyl)lysine
CRF: Chronic renal failure
CVD: Cardiovascular disease
DM: Diabetes mellitus
DN-HD: Hemodialysis patients with diabetic nephropathy
DPP-4: Dipeptidyl peptidase-4
3-DG: 3-Deoxyglucosone
3-DG-AGEs: 3-DG-derived AGEs
EC: Endothelial cells
ELISA: Enzyme-linked immunosorbent assay
Fru-AGEs: Fructose-derived AGEs
EPC: Endothelial progenitor cells
GA: Glyceraldehyde
Glu-AGEs: Glucose-derived AGEs
Glycer-AGEs: GA-derived AGEs
GO: Glyoxal
GO-AGEs: GO-derived AGEs
γ-GTP: γ-Glutamyl transpeptidase
HbA1c: Hemoglobin A1c
HCC: Hepatocellular carcinoma
HFCS: High-fructose corn syrup
HOME-IR: Homeostatic model assessment of insulin resistance
HSC: Hepatic stellate cells
IR: Insulin resistance
LSRD: Lifestyle-related diseases
MAGE: Mean amplitude of glycemic excursions
MCP-1: Monocyte chemoattractant protein-1
MGO: Methylglyoxal
MGO-AGEs: MGO-derived AGEs
NAFL: Non-alcoholic fatty liver
NAFLD: Non-alcoholic fatty liver disease
NASH: Non-alcoholic steatohepatitis
NBNC-HCC: Non-B or non-C HCC
OR: Odds ratio
RAGE: Receptor for AGEs
ROS: Reactive oxygen species
SSB: Sugar-sweetened beverages
TAGE: Toxic AGEs
TGF-β1: Transforming growth factor-β1
VCAM-1: Vascular cell adhesion molecule-1
VEGF: Vascular endothelial growth factor
WHO: World Health Organization
